# The Predictive Role of Radiomics in Breast Cancer Patients Imaged by [^18^F]FDG PET: Preliminary Results from a Prospective Cohort

**DOI:** 10.3390/diagnostics14202312

**Published:** 2024-10-17

**Authors:** Fabrizia Gelardi, Lara Cavinato, Rita De Sanctis, Gaia Ninatti, Paola Tiberio, Marcello Rodari, Alberto Zambelli, Armando Santoro, Bethania Fernandes, Arturo Chiti, Lidija Antunovic, Martina Sollini

**Affiliations:** 1 Department of Biomedical Sciences, Humanitas University, 20072 Pieve Emanuele, Italy; gelardi.fabrizia@hsr.it (F.G.); rita.de_sanctis@hunimed.eu (R.D.S.); paola.tiberio@cancercenter.humanitas.it (P.T.); alberto.zambelli@hunimed.eu (A.Z.); armando.santoro@cancercenter.humanitas.it (A.S.); 2 Faculty of Medicine and Surgery, Vita-Salute San Raffaele University, 20132 Milan, Italy; chiti.arturo@hsr.it (A.C.); sollini.martina@hsr.it (M.S.); 3 IRCCS San Raffaele Hospital, 20132 Milan, Italy; ninatti.gaia@hsr.it; 4 MOX, Department of Mathematics, Politecnico di Milano, 20133 Milan, Italy; lara.cavinato@polimi.it; 5 IRCCS Humanitas Research Hospital, 20089 Rozzano, Italy; marcello.rodari@humanitas.it (M.R.); bethania.fernandes@humanitas.it (B.F.); 6 School of Medicine and Surgery, University of Milano-Bicocca, 20900 Monza, Italy

**Keywords:** breast cancer, [^18^F]FDG, PET/CT, radiomics, pathologic complete response, neoadjuvant chemotherapy

## Abstract

Background: Recently, radiomics has emerged as a possible image-derived biomarker, predominantly stemming from retrospective analyses. We aimed to prospectively assess the predictive role of [^18^F]FDG-PET radiomics in breast cancer (BC). Methods: Patients affected by stage I–III BC eligible for neoadjuvant chemotherapy (NAC) staged with [^18^F]FDG-PET/CT were prospectively enrolled. The pathological response to NAC was assessed on surgical specimens. From each primary breast lesion, we extracted radiomic PET features and their predictive role with respect to pCR was assessed. Uni- and multivariate statistics were used for inference; principal component analysis (PCA) was used for dimensionality reduction. Results: We analysed 93 patients (53 HER2+ and 40 triple-negative (TNBC)). pCR was achieved in 44/93 cases (24/53 HER2+ and 20/40 TNBC). Age, molecular subtype, Ki67 percent, and stage could not predict pCR in multivariate analysis. In univariate analysis, 10 radiomic indices resulted in *p* < 0.1. We found that 3/22 radiomic principal components were discriminative for pCR. Using a cross-validation approach, radiomic principal components failed to discriminate pCR groups but predicted the stage (mean accuracy = 0.79 ± 0.08). Conclusions: This study shows the potential of PET radiomics for staging purposes in BC; the possible role of radiomics in predicting the pCR response to NAC in BC needs to be further investigated.

## 1. Introduction

Breast cancer (BC) is the most common cancer in women [1]. It is the leading cause of cancer-related death in women and the fourth leading cause when considering both sexes [2]. BC is characterized by significant disparities in early diagnosis, treatment, and management, which result in suboptimal outcomes worldwide. Effective management of BC, which remains a costly global health issue, critically depends on early detection. This cannot be overstated, and accessible, cost-effective treatment options, consisting of local versus systemic treatment [3], are mandatory. Neoadjuvant chemotherapy (NAC) is offered before surgery, as part of the standard of care, to patients with locally advanced HR+/HER2− BC, early HER2+ BC, or triple-negative BC (TNBC) [4]. Assessment of the pathologic response to NAC, which can be evaluated using different systems [5], is crucial in guiding clinical decisions and prognosis. Prediction of the pathologic response to NAC has the potential to limit exposure to unnecessary treatments, resulting in fewer side effects, thereby improving quality of life and ultimately reducing healthcare costs. Many models based on imaging, pathological, and clinical biomarkers—or their combination—have been developed and tested to proactively predict the response at an early stage of treatment or prior to the first cycle of NAC [6,7,8,9,10,11,12,13,14]. Although, theoretically, BC patients may benefit from these models that aim to improve outcomes, differences in molecular subtypes and NAC regimens, inconsistencies in acquisition protocols and types of examination (e.g., mammography, echo, [^18^F]FDG PET/CT, MRI, etc.), variables included in the models, and the systems used to assess the pathological response to NAC all represent inherent limitations. Moreover, the study design—which is largely retrospective—and the often-small sample size further affect the reliability of studies [15,16]. The present study aimed to assess the predictive role of [^18^F]FDG PET radiomics in a prospective cohort of BC patients eligible for NAC.

## 2. Materials and Methods

### 2.1. Population

Patients diagnosed with BC who had been referred to the Breast Unit at the IRCCS Humanitas Research Hospital were screened and invited to participate in this observational prospective trial. Inclusion criteria were female gender, age ≥ 18 years, and locally advanced histologically confirmed BC eligible for neoadjuvant treatment as per the standard of care. All patients signed an informed consent form before joining this study. We included in the present analyses all patients prospectively recruited from July 2019 to October 2022 who received a staging whole-body [^18^F]FDG PET/CT and underwent surgery after the end of NAC. Demographic data, histopathological tumour characteristics, and treatment response assessment were collected for each patient. Pathological treatment response in the breast surgical specimen removed after NAC, assessed according to Pinder et al. [17], was used as the reference standard. Additionally, the treatment response assessed by imaging was also recorded. All procedures were conducted in accordance with the Declaration of Helsinki and were approved by the IRCCS Humanitas Research Hospital Ethics Committee (protocol identifying number: ONC/OSS-02/2019).

### 2.2. PET Acquisition and Image Analyses

Whole-body images were acquired on an integrated PET/CT scanner (GE Discovery PET/CT 690, GE, Waukesha, WI, USA, Biograph, Siemens, Munich, Germany), according to the EANM guidelines [18], in fasting patients (at least 4 h) about one hour after an intravenous injection of about 6 MBq/kg [^18^F]FDG. Details about scanners and the acquisition protocol are provided in Supplementary Table S1.

For each patient, the primary breast lesion was semi-automatically segmented by an experienced nuclear medicine physician (LA) applying the 40% SUVmax threshold using LIFEx software (www.lifex.org [19], version 7.2.0, access date 1 May 2022). From the volume of interest (VOI), we extracted 71 first-order radiomic (morphological, intensity-based, histogram-based) features according to the Imaging Biomarkers Standardization Initiative (IBSI) [20], as detailed in Supplementary Table S1.

### 2.3. Statistical Analysis

Frequency tables and descriptive statistics were used to summarize patient characteristics. For further statistical analyses, we considered all patients with the HER2+ and TNBC subtypes, thus neglecting data from luminal A and luminal B cancers because of the low numerosity of the samples, which would confound the findings. The clinical variables considered for the analysis in addition to molecular subtypes included age, ki-67, ER and PgR expression levels, and lymph nodal status. According to the reference standard (pathological response to NAC), patients were divided into two categories: pathological complete response (pCR) and non-complete pathological response (non-pCR). Radiomic features were affected by episodes of missing values, in some cases leading to sparse vectors. To avoid deficiencies in further analyses, we imputed missing values using the HyperImpute method [21], an imputation framework for adaptively configuring column-wise imputation models and their hyperparameters (Supplementary Table S2). HyperImpute is a robust statistical method specifically designed to handle missing data, also in medical datasets. This approach automatically selects the most appropriate imputation model for each missing data type, ensuring accurate estimations. For instance, in cases where data could be predicted based on available values, the framework would fit a regression model to estimate the most probable value. We are confident that this imputation process did not affect our results in any significant way. Moreover, as the data were collected using two different scanners, we harmonized them for any differences arising from the diverse settings and hardware specifications using the ComBat method [22]. An initial exploratory analysis was performed on both clinical and radiomic variables by implementing univariate statistical tests so as to assess differences between the distributions of each variable in the responding, i.e., pCR, and non-responding, i.e., non-pCR, populations. Specifically, for numerical variables, Shapiro tests were used to assess the normality of the data and, accordingly, independent t-test and non-parametric test results were employed. For categorical variables, chi-squared tests on contingency tables were used to assess the significant associations between each of the variables and the response variable, pCR. In addition, radiomic features were also tested for significance with respect to the clinical stage of the disease. The distributions of each radiomic feature in the three populations of interest (Stage I, Stage II, Stage III) were compared with either a parametric or non-parametric ANOVA to highlight any differences between them. Furthermore, multivariate analysis was performed. First, the relevance of clinical variables was tested multivariately using logistic regression (LR). We fed an LR model with non-redundant clinical variables—e.g., age, Ki67 percent, subtype, and stage—to predict the response variable, pCR. The model was applied to the whole dataset to describe the joint significance between variables. P-values for each of the considered variables were accessed to evaluate the model. From a prediction point of view, an additional LR model was built and trained in a cross-validation fashion and the results were evaluated in terms of accuracy (mean ± SD), precision (mean ± SD), and recall (mean ± SD). Due to their high correlation and dimensionality, the radiomic features were reduced using principal component analysis (PCA), by keeping a number of principal components that explained 90% of the dataset variability. This reduced dataset was fed into two LR models to evaluate the joint significance of radiomic variables in terms of p-values for each of the considered variables. The response variables were pCR for the first model and stage for the second model. Alongside, two cross-validated LR models were trained and validated to quantify the predictive power of radiomics with respect to pCR and stage in terms of accuracy (mean ± SD), precision (mean ± SD), and recall (mean ± SD).

Ultimately, clinical features (age, Ki67 percent, subtype, stage) and radiomic features that were determined to be significant in multivariate testing were jointly fed into a cross-validated LR model to predict pCR. Results were assessed in terms of accuracy (mean ± SD), precision (mean ± SD), and recall (mean ± SD).

## 3. Results

In the analysis, we included 93 out of the 103 enrolled BC patients (53 HER2+, 40 triple-negative, and 2 luminal A and 8 luminal B), excluding those with luminal subtypes. A summary of the baseline patient characteristics is provided in Table 1.

**Figure 1:**
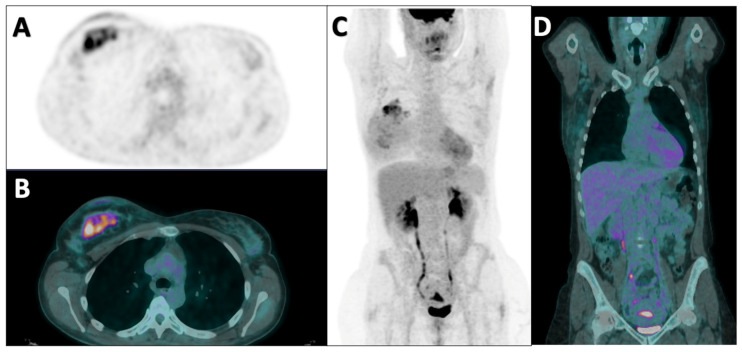
Staging [^18^F]FDG PET/CT in a 42-year-old patient with biopsy-proven invasive breast cancer, non-special type, G2, ER: 70%, PgR: 70%, Ki67: 25%. HER2+ (score 3+) and metastatic axillar lymph nodes at diagnosis (stage II). The patient underwent neoadjuvant chemotherapy with AC × 4 cycles, followed by Taxotere and Trastuzumab × 4 cycles. After the treatment, a mastectomy and sentinel lymph node biopsy were performed with histological evidence of rare foci of ductal carcinoma in situ, without an invasive component; sentinel lymph node was negative (ypTis N0). Axial PET (**A**), fused PET/CT (**B**), and coronal PET (**C**) images show a large non-homogenous area of increased tracer uptake in the upper quadrants of the right breast parenchyma. The coronal fused PET/CT image (**D**) shows multiple lymph nodes in the right axilla characterized by faint [^18^F]FDG uptake, in spite of being metastatic.

**Figure 2:**
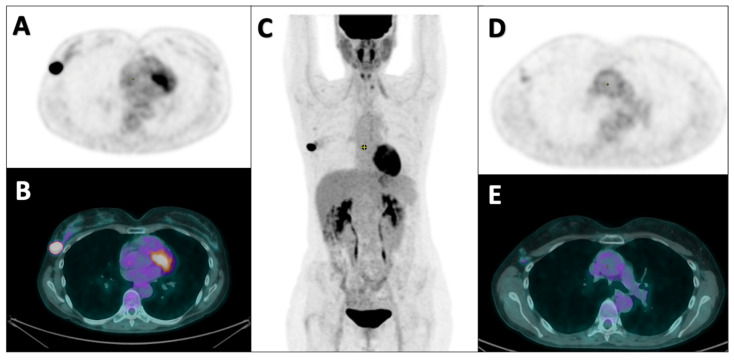
Staging [^18^F]FDG PET/CT in a 46-year-old patient with biopsy-proven invasive breast cancer, non-special type, G3, ER: 0%, PgR: 0%, Ki67: 50%. Her2+ (score 3+) and suspicious metastatic spread to axillary lymph nodes at diagnosis (stage II). The patient underwent neoadjuvant chemotherapy with AC × 4 cycles, followed by Taxotere and Trastuzumab × 4 cycles. After the treatment, a mastectomy and sentinel lymph node biopsy were performed with histological evidence of a single 12 mm focus of invasive breast carcinoma, non-special type, G3, sentinel lymph node negative (ypT1cN0 (sn)). Axial PET (**A**), fused PET/CT (**B**), and coronal PET (**C**) images show a focal area of increased pathological tracer uptake in the upper external right breast quadrant. Axial PET (**D**) and fused PET/CT (**E**) images show two sub-centimetric lymph nodes with increased [^18^F]FDG uptake in the right axillary region, suspected for metastases.

Figure 1 and Figure 2 show examples of two patients enrolled in this study.

**Figure 3:**
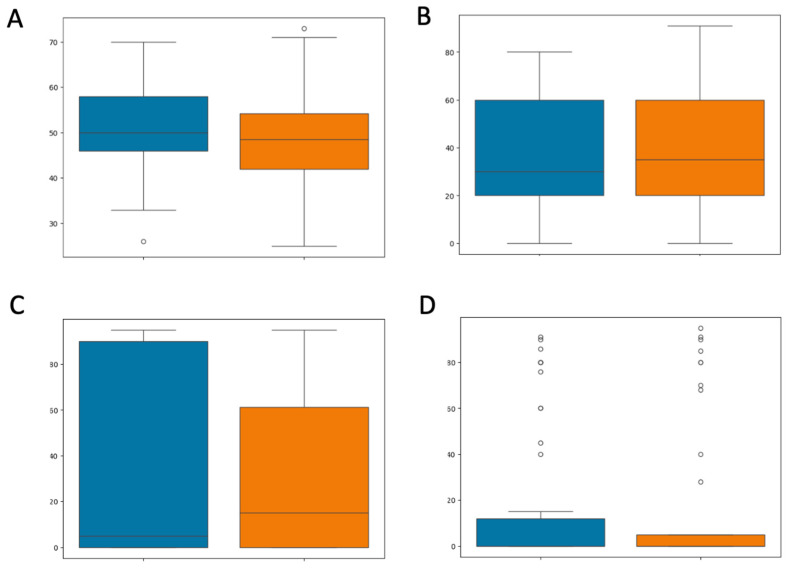
Boxplot of numerical variables according to NAC response assessment. Responding (i.e., pCR = 1) and non-responding (i.e., non-pCR = 0) classes are presented in orange and blue, respectively. Age (**A**), ki67 (**B**), ER (**C**), and PgR (**D**).

Supplementary Tables S3 and S4 summarize the statistics for the numerical and categorical variables, respectively. None of the clinical variables were significant when stratifying responding and non-responding patients. Among the numerical variables, the *p*-values of the tests for age, Ki67, ER, and PgR were 0.2259, 0.3395, 0.4914, and 0.4989, respectively (Figure 3).

The tests on the categorical variables of BC subtype, stage, and the presence of lymph nodes resulted in *p* = 0.8093, *p* = 0.4362, and *p* = 0.1594, highlighting no association with response.

Few radiomic variables showed significance in univariate testing. Specifically, by stratifying patients according to pCR response (Supplementary Table S5), 10/71 features were found to be significant, namely Centre-Of-Mass Shift (morphological), Variance, Median, Maximum Grey Level, Range, Quartile Coefficient Of Dispersion, Area Under Curve, Root Mean Square (intensity-based), Root Mean Square, and the Minimum Histogram Gradient (intensity histogram). Stratifying by the stage of the patients’ disease, 10/71 features, different from the ones above, displayed a p-value lower than 0.1 (Supplementary Table S6). These features are Surface-To-Volume Ratio, Compactness1, Compactness2, Sphericity (morphological), Kurtosis, Coefficient Of Variation (intensity-based), Intensity Histogram Standard Deviation, Intensity Histogram Coefficient Of Variation, Root Mean Square, and Maximum Histogram Gradient (intensity histogram).

The LR model applied to the whole clinical dataset confirmed a poor association of the clinical features with response. Table 2 displays the coefficients (and CI 95%) and the p-values for each of the clinical variables in the model.

The cross-validated LR model fed with clinical variables showed poor predictive power, as highlighted by the following prediction indexes: accuracy 0.4178 ± 0.0932; precision 0.4288 ± 0.1783; recall 0.4178 ± 0.0932.

The PCA kept the first 22 principal components that explained 90% of the dataset variability. Table 3 and Table 4 display the results for the LR models applied to the whole PCA-reduced radiomic dataset for predicting pCR and stage, respectively. Specifically, we present the coefficients (and CI 95%) and the *p*-values of each of the radiomic principal components in the models.

The cross-validated LR model predicting pCR returned an accuracy of 0.4975 ± 0.1084, a precision of 0.5213 ± 0.1154, and a recall of 0.4975 ± 0.1084. The cross-validated LR model predicting the stage resulted in 0.7914 ± 0.0761 for accuracy, 0.7457 ± 0.1227 for precision, and 0.7914 ± 0.0761 for recall.

Lastly, the cross-validated LR models fed with both clinical and radiomic features displayed an accuracy of 0.4663 ± 0.0976, a precision of 0.5002 ± 0.1183, and a recall of 0.4663 ± 0.0976.

## 4. Discussion

In this prospective cohort of BC patients, none of the variables tested were able to predict the treatment response to NAC. Specifically, the clinical model—including factors known to be prognostically relevant, such as molecular subtype and stage—resulted in a poor predictive performance (mean accuracy = 0.42 ± 0.09), and radiomics did not perform significantly better (mean accuracy = 0.50 ± 0.11). Besides the “negativity” of these results, the lack of a statistically significant correlation between at least one radiomic feature and NAC response was greatly unexpected, since 92% of articles published on this topic in the last 15 years have reported such a correlation [15]. Moreover, beyond (and before) radiomics, SUV-derived parameters extracted from [^18^F]FDG images have been suggested to be predictive biomarkers correlated to the response to NAC in many tumour types [23,24,25], including BCa [26]. However, radiomic features extracted from the primary BC tumour at baseline [^18^F]FDG PET/CT accurately predicted the stage (mean accuracy = 0.79 ± 0.08). Although our findings were more disappointing than expected, as we aimed to predict the treatment response to NAC, the ability to accurately predict the stage with features extracted from the primary tumour may offer great insights. Indeed, survival is stage-dependent. Stage is a key element in decision-making, and it is proportional to patient management expenses. According to current guidelines [4], the minimum workup for staging BC includes a chest and abdomen CT plus a bone scan. Although [^18^F]FDG PET/CT is not routinely recommended, it can be useful in the case of inconclusive conventional imaging results and in high-risk patients, where it can replace CT and bone scintigraphy [4,27,28]. Certainly, echo is of high value for the clinical assessment of lymph nodal status at diagnosis, although the risk of nodal involvement in patients with negative preoperative axillary ultrasound is not negligible, thereby confirming the validity of a lymph node sentinel biopsy [29,30]. Additionally, the assessment of internal mammary nodal involvement represents an issue [31]. MRI presents a high negative predictive value in excluding pN2-N3 [30]. A recent study demonstrated that [^18^F]FDG PET/MR outperformed CT and MRI in detecting nodal involvement in a patient-based analysis and in a lesion-based analysis [32]. The value of [^18^F]FDG PET/MR in staging BC has also been outlined by some recent meta-analyses [33,34]. However, the scarce availability of the technique is per se a constraint. Therefore, the possibility to objectively predict the clinical stage with baseline [^18^F]FDG PET/CT by extracting radiomic features from the primary tumour might certainly have a positive impact on BC management, helping to tailor personalized approaches, potentially sparing patients from unnecessary treatments and associated side effects, and reducing costs. Implementing such a predictive model in clinical practice could streamline decision-making processes and enhance the efficiency of BC management, potentially leading to improved survival rates and quality of life for patients.

Despite its promising results, our study had some limitations. Firstly, we presented a preliminary analysis. Further evaluation, including additional parameters obtained from this patient population, is ongoing. However, the study design with prospective data collection ensured high-quality data. Moreover, to increase robustness we harmonized features and imputed missing radiomic data. It is worth considering that the relatively modest results observed in our analysis could be due to the exclusion of higher-order radiomic features, which have been shown to carry more predictive power in other studies and in other oncological contexts. However, higher-order statistics often require specific conditions, such as a minimum number of voxels or specific imaging constraints, which may not always be feasible in breast cancer studies, as in our case. Indeed, missing data for radiomic feature extraction are an inherent limitation of the method, as LIFEx calculates all radiomic features only for volumes of interest (VOIs) greater than 64 voxels. For VOIs that do not meet the minimum size criterion, LIFEx extracts only first-order parameters, as higher-order features would not allow for reliable calculation. Overall, first-order statistics formed the basis of our analysis, ensuring that the missing data did not substantially affect the integrity of our study. Secondly, as mentioned above, radiomics is affected by some methodological issues. However, we performed data harmonization to address this question. Thirdly, the use of PCA to reduce dimensionality ensured the retention of most of the information, which explained 90% of variability in the original radiomic dataset.

## 5. Conclusions

Radiomic features extracted from baseline [^18^F]FDG PET/CT failed to predict the NAC treatment response in this preliminary analysis of a prospective cohort of BC patients. However, radiomic features extracted from the primary tumour accurately predicted the clinical stage of the disease. This approach could improve BC management, optimizing treatment strategies, positively impacting patient outcomes, and reducing healthcare costs.

## Figures and Tables

**Table 1:** Baseline patient characteristics.

	Population (*n* = 93)	
	*n*	%
**Age, years**		
Mean (±SD)	49 (±10.04)	
**Molecular subtype**		
HER2+	53	57%
TNBC	40	43%
**Ki67, %**		
Mean (±SD)	39.98 (±25.40)	
Median (range)	35 (0–91)	
**Oestrogen receptor, %**		
Mean (±SD)	31.18 (±33.61)	
Median (range)	10 (0–95)	
**Progesterone receptor, %**		
Mean (±SD)	16.97 (±31.41)	
Median (range)	0 (0–96)	
**Clinical stage at diagnosis**		
I	4	4%
II	76	82%
III	13	14%
**Clinical lymph node status**		
Negative	51	55%
Positive	42	45%
**PET/CT scanner**		
Biograph Siemens	39	42%
GE Discovery PET/CT 690	54	58%
**Response to NAC**		
Complete	49	53%
Non-complete	44	47%
**Treatment response assessment by imaging**		
Complete	44	47%
Non-complete	49	53%

SD—standard deviation; HER2+—human epidermal growth factor receptor positive; TNBC—triple-negative breast cancer; PET/CT—positron emission tomography/computed tomography; NAC—neoadjuvant chemotherapy.

**Table 2:** Variables included in logistic regression model for prediction of pathological response.

Variable	Coefficient	CI Lower Bound	CI Upper Bound	*p*-Value
Age	−0.0240	−0.068	0.020	0.285
Ki67 percent	−0.0078	−0.025	0.010	0.384
HER2	2.6936	−0.869	6.256	0.138
TNBC	2.9422	−0.684	6.568	0.112
Stage	−0.6752	−1.716	0.366	0.204

CI: confidence interval; HER2: human epidermal growth factor receptor; TNBC: triple-negative breast cancer.

**Table 3:** Logistic regression model applied on PCA-reduced radiomic dataset for prediction of pathological response.

Variable	Coefficient	CI Lower Bound	CI Upper Bound	*p*-Value
x1	−0.0401	−0.182	0.102	0.581
x2	0.0248	−0.136	0.186	0.763
x3	−0.0140	−0.237	0.209	0.902
x4	−0.1590	−0.455	0.137	0.292
x5	−0.0252	−0.301	0.250	0.858
x6	0.1593	−0.178	0.496	0.354
x7	−0.2742	−0.666	0.118	0.171
x8	−0.2830	−0.747	0.181	0.232
x9	0.0136	−0.390	0.417	0.947
x10	0.2369	−0.326	0.800	0.409
x11	0.1690	−0.310	0.648	0.489
x12	0.5320	−0.111	1.175	**0.105**
x13	−0.4323	−0.962	0.098	0.110
x14	0.7864	−0.031	1.604	**0.059**
x15	0.5682	−0.149	1.286	0.121
x16	−0.2282	−0.865	0.408	0.482
x17	0.7658	−0.158	1.689	**0.104**
x18	0.2125	−0.558	0.983	0.589
x19	−0.1954	−0.939	0.548	0.606
x20	0.0483	−0.700	0.796	0.899
x21	−0.9392	−2.162	0.284	0.132
x22	−0.9904	−2.311	0.331	0.142

CI: confidence interval.

**Table 4:** Logistic regression model applied on PCA-reduced radiomic dataset for prediction of disease stage.

Variable	Coefficient	CI Lower Bound	CI Upper Bound	*p*-Value
Stage = II				
x1	−0.0063	−0.143	0.131	0.929
x2	−0.0939	−0.286	0.098	0.338
x3	−0.4111	−0.736	−0.086	**0.013**
x4	−0.2153	−0.565	0.135	0.228
x5	0.1033	−0.189	0.396	0.488
x6	0.0455	−0.293	0.384	0.792
x7	−0.2018	−0.629	0.226	0.355
x8	−0.5497	−1.018	−0.082	**0.021**
x9	0.2465	−0.184	0.677	0.262
x10	−1.0194	−1.881	−0.158	**0.020**
x11	−0.5763	−1.172	0.020	**0.058**
x12	−0.3706	−1.107	0.366	0.324
x13	−0.3248	−0.898	0.248	0.267
x14	0.0161	−0.605	0.637	0.959
x15	0.1510	−0.514	0.816	0.656
x16	−0.2182	−0.982	0.546	0.576
x17	0.2430	−0.458	0.944	0.497
x18	0.7428	−0.102	1.588	**0.085**
x19	−0.0625	−0.812	0.687	0.870
x20	−0.2221	−1.059	0.615	0.603
x21	−0.5705	−1.532	0.391	0.245
x22	0.5229	−0.424	1.469	0.279
Stage = III				
x1	0.0335	−0.103	0.170	0.631
x2	−0.0138	−0.183	0.155	0.873
x3	0.0902	−0.155	0.336	0.472
x4	0.0068	−0.299	0.313	0.965
x5	−0.0260	−0.315	0.263	0.860
x6	−0.2672	−0.649	0.114	0.170
x7	0.0517	−0.297	0.401	0.772
x8	0.0948	−0.270	0.460	0.611
x9	−0.2550	−0.690	0.180	0.250
x10	0.2154	−0.377	0.808	0.476
x11	0.2275	−0.277	0.732	0.377
x12	−0.2726	−0.927	0.381	0.414
x13	0.0485	−0.552	0.649	0.874
x14	−0.0548	−0.779	0.669	0.882
x15	0.0976	−0.586	0.781	0.780
x16	0.1016	−0.616	0.819	0.781
x17	−0.5306	−1.516	0.454	0.291
x18	0.1759	−0.591	0.942	0.653
x19	−0.0450	−0.880	0.790	0.916
x20	−0.2927	−1.157	0.572	0.507
x21	0.1838	−0.780	1.147	0.709
x22	−0.2705	−1.209	0.668	0.572

## Data Availability

This manuscript represents valid work, and neither this manuscript nor one with substantially similar content under the same authorship has been published or is being considered for publication elsewhere. All the original patient DICOM files are stored in the institutional PACS. The calculations of the features are stored in the repository and available upon request (https://zenodo.org/records/12799495, created on 23 July 2024).

## Supplementary Materials

The following supporting information can be downloaded at https://www.mdpi.com/article/10.3390/diagnostics14202312/s1. Table S1: Report on image processing and image biomarker extraction; Table S2: Number of missing values per feature; Table S3: Descriptive statistics of the numerical variables stratified into groups based on the pathological response; Table S4: Contingency statistics of the categorical variables based on the binary response (pCR) and p-value of the chi-square test of the associations (pCR-variable); Table S5: Univariate testing of radiomic features according to pCR response; Table S6: Univariate testing of radiomic features according to disease stage.
