# Supplementary material for: The Predictive Role of Radiomics in Breast Cancer Patients Imaged by [18F]FDG PET: Preliminary Results from a Prospective Cohort
Source: Diagnostics (Basel). 2024 Oct 17;14(20):2312. doi: 10.3390/diagnostics14202312 (PMC11506751; doi:10.3390/diagnostics14202312)
Supplement: Supplementary file 1 [file diagnostics-14-02312-s001.zip › diagnostics-3147768-supplementary.pdf]

Supplementary material

The predictive role of radiomics in breast cancer patients imaged by [^{18}F]FDG PET: preliminary results from a prospective cohort

Fabrizia Gelardi[1,2,3Ψ],Lara Cavinato[4Ψ], Rita De Sanctis[1,5], Gaia Ninatti[3,6], Paola Tiberio[1,5], Marcello Rodari[5], Alberto Zambelli[1,5], Armando Santoro[1,5], Bethania Fernandes[5], Arturo Chiti [2,3], Lidija Antunovic[2*], Martina Sollini[2,3]

[1]Humanitas University, Pieve Emanuele, Italy
[2]Vita-Salute San Raffaele University, Milan, Italy
[3]IRCCS San Raffaele Hospital, Milan, Italy
[4]MOX, Department of Mathematics, Politecnico di Milano, Milano, Italy
[5]IRCCS Istituto Clinico Humanitas, Rozzano, Italy
[6]University of Milano-Bicocca, Monza, Italy
[Ψ]These authors contributed equally to this paper.
* Correspondence: antunovic.lidija@hsr.it

Supplementary Table S1. Report on image processing and image biomarker extraction.

Acquisition and reconstruction

**Acquisition parameters**	**Biograph – Siemens**		**Discovery 690 – General Electric**	
	PET	CT	PET	CT
**^{18}F-FDG activity (MBq)***	350–550	–	350–550	–
**Min/bed position**	2.5	–	2	–
**Crystal**	LSO	–	LYSO	–
**Reconstruction**	Iterative	–	Iterative, TOF Sharp IR	–
**Matrix (pixels)**	128×128	512×512	256×256	512×512
**Resolution (mm)**	5.3×5.3	0.98×0.98	2.73×2.73	1.37×1.37
**Slice thickness (mm)**	2.0	4.0	3.27	3.27
**Slices**	–	6	–	64
**Voltage (kV)**	–	130	–	140
**Tube current (mA)**	–	95	–	140
**Reconstruction**		–	Iterative, TOF Sharp IR	–

*Administered activity was calculated according to the European Association of Nuclear Medicine (EANM) guidelines 2.0 (Boellaard et al., 2014)

Approach	The images were analysed as a volume (3D).

Process structure	Image acquisition -> reconstruction -> anonymisation -> segmentation -> export -> texture analysis -> feature calculation report
Software	LIFEx version 7.2.0<br>(IMIV, CEA, Inserm, CNRS, Univ. Paris-Sud, Université Paris Saclay, CEA-SHFJ, 91400, Orsay, France)
Data availability	All the original patient DICOM files are stored in the institutional PACS. The calculations of the features are stored in the repository and available at request (https://zenodo.org/records/12799495)
**Data conversion**	
Procedure	None
**Image post-acquisition processing**	
Procedure	None
**Segmentation**	
ROI	The volume of interest (VOI) included the primary tumour lesion. Textural features were calculated on PET images within the same VOI.
Procedure	The ROIs were (semi-)automatically defined on PET images with a threshold of 40% of the maximum standardised uptake value (SUV_{max}) using LIFEx.
**Interpolation**	
Voxel dimensions	Not applicable
Image interpolation method	Not applicable
Image intensity rounding	Not applicable
ROI interpolation method	Not applicable
ROI partial volume	Not applicable
**Re-segmentation**	
ROI mask criteria	None
**Discretisation**	
Discretisation method	PET: 64 bins from 0 to 25
**Feature calculation**	

Feature set	**MORPHOLOGICAL**<br>Volume<br>ApproximateVolume<br>voxelsCounting<br>SurfaceArea<br>SurfaceToVolumeRatio<br>Compacity<br>Compactness1<br>Compactness2<br>SphericalDisproportion<br>Sphericity<br>CentreOfMassShift<br>Maximum3DDiameter<br>IntegratedIntensity<br>**INTENSITY-BASED**<br>Variance(SUVbw)<br>Skewness(SUVbw)<br>Kurtosis(SUVbw)<br>Median(SUVbw)<br>MinimumGreyLevel(SUVbw)<br>StandardDeviation(SUVbw)<br>MaximumGreyLevel(SUVbw)<br>InterquartileRange(SUVbw)<br>Range(SUVbw)<br>MeanAbsoluteDeviation(SUVbw)<br>RobustMeanAbsoluteDeviation(SUVbw)<br>MedianAbsoluteDeviation(SUVbw)<br>CoefficientOfVariation(SUVbw)<br>QuartileCoefficientOfDispersion(SUVbw)<br>AreaUnderCurveCsh(SUVbw)<br>Energy(SUVbw)<br>RootMeanSquare(SUVbw)<br>TotalLesionGlycolysis(SUVbw)<br>IntensityPeakDiscretizedVolumeSought(0.5mL)(mL)<br>GlobalIntensityPeak(0.5mL)(SUVbw)<br>IntensityPeakDiscretizedVolumeSought(1mL)(mL)<br>GlobalIntensityPeak(1mL)(SUVbw)<br>LocalIntensityPeak(SUVbw)<br>**INTENSITY-HISTOGRAM**<br>IntensityHistogramMean(SUVbw)<br>IntensityHistogramVariance(SUVbw)<br>IntensityHistogramSkewness(SUVbw)<br>IntensityHistogramKurtosis(SUVbw)<br>IntensityHistogramMedian(SUVbw)<br>IntensityHistogramMinimumGreyLevel(SUVbw)<br>IntensityHistogram10thPercentile(SUVbw)<br>IntensityHistogram25thPercentile(SUVbw)<br>IntensityHistogram50thPercentile(SUVbw)<br>IntensityHistogram75thPercentile(SUVbw)

	IntensityHistogram90thPercentile(SUVbw)<br>IntensityHistogramStandardDeviation(SUVbw)<br>IntensityHistogramMaximumGreyLevel(SUVbw)<br>IntensityHistogramMode(SUVbw)<br>IntensityHistogramInterquartileRange(SUVbw)<br>IntensityHistogramRange(SUVbw)<br>IntensityHistogramMeanAbsoluteDeviation(SUVbw)<br>IntensityHistogramRobustMeanAbsoluteDeviation(SUVbw)<br>IntensityHistogramMedianAbsoluteDeviation(SUVbw)<br>IntensityHistogramCoefficientOfVariation(SUVbw)<br>IntensityHistogramQuartileCoefficientOfDispersion(SUVbw)<br>IntensityHistogramEntropyLog10(SUVbw)<br>IntensityHistogramEntropyLog2(SUVbw)<br>AreaUnderCurveCsh(SUVbw)<br>Uniformity(SUVbw)<br>RootMeanSquare(SUVbw)<br>MaximumHistogramGradient(SUVbw)<br>MaximumHistogramGradientGreyLevel(SUVbw)<br>MinimumHistogramGradient(SUVbw)<br>MinimumHistogramGradientGreyLevel(SUVbw)<br>IntensityPeakDiscretizedVolumeSought(0.5mL)(mL)<br>GlobalIntensityPeak(0.5mL)<br>IntensityPeakDiscretizedVolumeSought(1mL)(mL)<br>GlobalIntensityPeak(1mL)(SUVbw)<br>LocalIntensityPeak(SUVbw)
Feature parameters	The parameters were calculated according to the formulas described in the software manual (LIFEx version 7.2.0)<br>(IMIV, CEA, Inserm, CNRS, Univ. Paris-Sud, Université Paris Saclay, CEA-SHFJ, 91400, Orsay, France)
Standardisation	Not performed

Supplementary Table S2: number of missing values per feature

**Clinical feature**	
age	0
BC subtype	0
Ki67_percent	0
ER	0
PgR	0
Stage	0
Ln : 0 =neg, 1=pos	0
RISPOSTA pCR (1) / non PCR (0)	0
**Radiomic feature**	
MORPHOLOGICAL_Volume(IBSI:RNU0)	0

MORPHOLOGICAL_ApproximateVolume(IBSI:YEKZ)	0
MORPHOLOGICAL_voxelsCounting(IBSI:No)	0
MORPHOLOGICAL_SurfaceArea(IBSI:C0JK)	0
MORPHOLOGICAL_SurfaceToVolumeRatio(IBSI:2PR5)	0
MORPHOLOGICAL_Compacity(IBSI:No)	0
MORPHOLOGICAL_Compactness1(IBSI:SKGS)	0
MORPHOLOGICAL_Compactness2(IBSI:BQWJ)	0
MORPHOLOGICAL_SphericalDisproportion(IBSI:KRCK)	0
MORPHOLOGICAL_Sphericity(IBSI:QCFX)	0
MORPHOLOGICAL_CentreOfMassShift(IBSI:KLMA)	0
MORPHOLOGICAL_Maximum3DDiameter(IBSI:L0JK)	0
MORPHOLOGICAL_IntegratedIntensity(IBSI:99N0)	0
INTENSITY-BASED_Variance(SUVbw)IBSI:ECT3	0
INTENSITY-BASED_Skewness(SUVbw)IBSI:KE2A	0
INTENSITY-BASED_Kurtosis(SUVbw)IBSI:IPH6	0
INTENSITY-BASED_Median(SUVbw)IBSI:Y12H	0
INTENSITY-BASED_MinimumGreyLevel(SUVbw)IBSI:1GSF	0
INTENSITY-BASED_StandardDeviation(SUVbw)IBSI:No	0
INTENSITY-BASED_MaximumGreyLevel(SUVbw)IBSI:84IY	0
INTENSITY-BASED_InterquartileRange(SUVbw)IBSI:SALO	0
INTENSITY-BASED_Range(SUVbw)IBSI:2OJQ	0
INTENSITY-BASED_MeanAbsoluteDeviation(SUVbw)IBSI:4FUA	0
INTENSITY-BASED_RobustMeanAbsoluteDeviation(SUVbw)IBSI:1128	0
INTENSITY-BASED_MedianAbsoluteDeviation(SUVbw)IBSI:N72L	0
INTENSITY-BASED_CoefficientOfVariation(SUVbw)IBSI:7TET	0
INTENSITY-BASED_QuartileCoefficientOfDispersion(SUVbw)IBSI:9S40	0
INTENSITY-BASED_AreaUnderCurveCsh(SUVbw)IBSI:No	0
INTENSITY-BASED_Energy(SUVbw)IBSI:N8CA	0
INTENSITY-BASED_RootMeanSquare(SUVbw)IBSI:5ZWQ	0
INTENSITY-BASED_TotalLesionGlycolysis(SUVbw)IBSI:No	0
LOCAL_INTENSITY_BASED_IntensityPeakDiscretizedVolumeSought(0.5mL)(mL)IBSI:No	8
LOCAL_INTENSITY_BASED_GlobalIntensityPeak(0.5mL)(SUVbw)IBSI:No	2
LOCAL_INTENSITY_BASED_IntensityPeakDiscretizedVolumeSought(1mL)(mL)IBSI:No	8
LOCAL_INTENSITY_BASED_GlobalIntensityPeak(1mL)(SUVbw)IBSI:0F91	1
LOCAL_INTENSITY_BASED_LocalIntensityPeak(SUVbw)IBSI:VJGA	8
INTENSITY-HISTOGRAM_IntensityHistogramMean(SUVbw)IBSI:X6K6	0
INTENSITY-HISTOGRAM_IntensityHistogramVariance(SUVbw)IBSI:CH89	0
INTENSITY-HISTOGRAM_IntensityHistogramSkewness(SUVbw)IBSI:88K1	1
INTENSITY-HISTOGRAM_IntensityHistogramKurtosis(SUVbw)IBSI:C3I7	1
INTENSITY-HISTOGRAM_IntensityHistogramMedian(SUVbw)IBSI:WIFQ	0

INTENSITY-HISTOGRAM_IntensityHistogramMinimumGreyLevel(SUVbw)IBSI:1PR8	0
INTENSITY-HISTOGRAM_IntensityHistogram10thPercentile(SUVbw)IBSI:GPMT	0
INTENSITY-HISTOGRAM_IntensityHistogram25thPercentile(SUVbw)IBSI:No	0
INTENSITY-HISTOGRAM_IntensityHistogram50thPercentile(SUVbw)IBSI:No	0
INTENSITY-HISTOGRAM_IntensityHistogram75thPercentile(SUVbw)IBSI:No	0
INTENSITY-HISTOGRAM_IntensityHistogram90thPercentile(SUVbw)IBSI:OZ0C	0
INTENSITY-HISTOGRAM_IntensityHistogramStandardDeviation(SUVbw)IBSI:No	0
INTENSITY-HISTOGRAM_IntensityHistogramMaximumGreyLevel(SUVbw)IBSI:3NCY	0
INTENSITY-HISTOGRAM_IntensityHistogramMode(SUVbw)IBSI:AMMC	0
INTENSITY-HISTOGRAM_IntensityHistogramInterquartileRange(SUVbw)IBSI:WR0O	0
INTENSITY-HISTOGRAM_IntensityHistogramRange(SUVbw)IBSI:5Z3W	0
INTENSITY-HISTOGRAM_IntensityHistogramMeanAbsoluteDeviation(SUVbw)IBSI:D2ZX	0
INTENSITY-HISTOGRAM_IntensityHistogramRobustMeanAbsoluteDeviation(SUVbw)IBSI:WRZB	0
INTENSITY-HISTOGRAM_IntensityHistogramMedianAbsoluteDeviation(SUVbw)IBSI:4RNL	0
INTENSITY-HISTOGRAM_IntensityHistogramCoefficientOfVariation(SUVbw)IBSI:CWYJ	0
INTENSITY-HISTOGRAM_IntensityHistogramQuartileCoefficientOfDispersion(SUVbw)IBSI:SLWD	0
INTENSITY-HISTOGRAM_IntensityHistogramEntropyLog10(SUVbw)IBSI:No	0
INTENSITY-HISTOGRAM_IntensityHistogramEntropyLog2(SUVbw)IBSI:TLU2	0
INTENSITY-HISTOGRAM_AreaUnderCurveCsh(SUVbw)IBSI:No	0
INTENSITY-HISTOGRAM_Uniformity(SUVbw)IBSI:BJ5W	0
INTENSITY-HISTOGRAM_RootMeanSquare(SUVbw)IBSI:No	0
INTENSITY-HISTOGRAM_MaximumHistogramGradient(SUVbw)IBSI:12CE	0
INTENSITY-HISTOGRAM_MaximumHistogramGradientGreyLevel(SUVbw)IBSI:8E6O	0
INTENSITY-HISTOGRAM_MinimumHistogramGradient(SUVbw)IBSI:VQB3	0
INTENSITY-HISTOGRAM_MinimumHistogramGradientGreyLevel(SUVbw)IBSI:RHQZ	0
LOCAL_INTENSITY_HISTOGRAM_IntensityPeakDiscretizedVolumeSought(0.5mL)(mL)IBSI:No	8
LOCAL_INTENSITY_HISTOGRAM_GlobalIntensityPeak(0.5mL)(SUVbw)IBSI:No	2
LOCAL_INTENSITY_HISTOGRAM_IntensityPeakDiscretizedVolumeSought(1mL)(mL)IBSI:No	8
LOCAL_INTENSITY_HISTOGRAM_GlobalIntensityPeak(1mL)(SUVbw)IBSI:No	1
LOCAL_INTENSITY_HISTOGRAM_LocalIntensityPeak(SUVbw)IBSI:No	8

Supplementary Table S3. Descriptive statistics of the numerical variables stratified into groups based on the pathological response.

	pCR	count	mean	std	min	25%	50%	75%	max	p-values
Age	0	49.00	50.10	9.81	26.00	46.00	50.00	58.00	70.00	0.2259
	1	44.00	48.52	10.33	25.00	42.00	48.50	54.25	73.00	
Ki67	0	49.00	40.68	25.36	0.00	20.00	30.00	60.00	80.00	0.3395
	1	44.00	38.47	25.85	0.00	20.00	35.00	60.00	91.00	

ER	0	49.00	33.65	41.55	0.00	0.00	5.00	90.00	95.00	0.4914
	1	44.00	33.47	36.42	0.00	0.00	15.00	61.25	95.00	
PgR	0	49.00	16.95	30.96	0.00	0.00	0.00	12.00	91.00	0.4989
	1	44.00	16.97	32.25	0.00	0.00	0.00	5.00	95.00	
ER: estrogen receptor; max: maximum; min: minimum; pCR: pathological complete response; PgR: progesteron receptor; std: standard										

Supplementary Table S4. Contingency statistics of the categorical variables based on the binary response (pCR) and p-value of the chi-square test of the associations (pCR-variable).

	non-pCR (=0)	pCR (=1)	p-value
BC subtype			
HER2+	29	24	0.8093
TNBC	20	20	
Stage			
I	2	2	0.4362
II	38	38	
III	9	4	
Lymph node status			
Negative	23	28	0.1594
Positive	26	16	
pCR: pathological complete response; BC: breast cancer; HER2+ human epidermal growth factor receptor positive; TNBC: triple negative breast cancer			

Supplementary Table S5. Univariate testing of radiomic features according to pCR response.

Variabile	p-val
MORPHOLOGICAL_Volume(IBSI:RNU0)	0.3025
MORPHOLOGICAL_ApproximateVolume(IBSI:YEKZ)	0.1386
MORPHOLOGICAL_voxelsCounting(IBSI:No)	0.4101
MORPHOLOGICAL_SurfaceArea(IBSI:C0JK)	0.3753
MORPHOLOGICAL_SurfaceToVolumeRatio(IBSI:2PR5)	0.1349
MORPHOLOGICAL_Compacity(IBSI:No)	0.4622
MORPHOLOGICAL_Compactness1(IBSI:SKGS)	0.1435
MORPHOLOGICAL_Compactness2(IBSI:BQWJ)	0.1140
MORPHOLOGICAL_SphericalDisproportion(IBSI:KRCK)	0.2127
MORPHOLOGICAL_Sphericity(IBSI:QCFX)	0.1555
MORPHOLOGICAL_CentreOfMassShift(IBSI:KLMA)	0.0933
MORPHOLOGICAL_Maximum3DDiameter(IBSI:L0JK)	0.3225
MORPHOLOGICAL_IntegratedIntensity(IBSI:99N0)	0.1470
INTENSITY-BASED_Variance(SUVbw)IBSI:ECT3	0.0950
INTENSITY-BASED_Skewness(SUVbw)IBSI:KE2A	0.1543
INTENSITY-BASED_Kurtosis(SUVbw)IBSI:IPH6	0.2071
INTENSITY-BASED_Median(SUVbw)IBSI:Y12H	0.0803
INTENSITY-BASED_MinimumGreyLevel(SUVbw)IBSI:1GSF	0.3583
INTENSITY-BASED_StandardDeviation(SUVbw)IBSI:No	0.1726
INTENSITY-BASED_MaximumGreyLevel(SUVbw)IBSI:84IY	0.0388
INTENSITY-BASED_InterquartileRange(SUVbw)IBSI:SALO	0.3250

INTENSITY-BASED_Range(SUVbw)IBSI:2OJQ	0.0539
INTENSITY-BASED_MeanAbsoluteDeviation(SUVbw)IBSI:4FUA	0.4452
INTENSITY-BASED_RobustMeanAbsoluteDeviation(SUVbw)IBSI:1128	0.4939
INTENSITY-BASED_MedianAbsoluteDeviation(SUVbw)IBSI:N72L	0.3571
INTENSITY-BASED_CoefficientOfVariation(SUVbw)IBSI:7TET	0.2302
INTENSITY-BASED_QuartileCoefficientOfDispersion(SUVbw)IBSI:9S40	0.0846
INTENSITY-BASED_AreaUnderCurveCsh(SUVbw)IBSI:No	0.0944
INTENSITY-BASED_Energy(SUVbw)IBSI:N8CA	0.2460
INTENSITY-BASED_RootMeanSquare(SUVbw)IBSI:5ZWQ	0.0541
INTENSITY-BASED_TotalLesionGlycolysis(SUVbw)IBSI:No	0.2229
LOCAL_INTENSITY_BASED_IntensityPeakDiscretizedVolumeSought(0.5mL)(mL)IBSI:No	0.1986
LOCAL_INTENSITY_BASED_GlobalIntensityPeak(0.5mL)(SUVbw)IBSI:No	0.1628
LOCAL_INTENSITY_BASED_IntensityPeakDiscretizedVolumeSought(1mL)(mL)IBSI:No	0.1986
LOCAL_INTENSITY_BASED_GlobalIntensityPeak(1mL)(SUVbw)IBSI:0F91	0.1316
LOCAL_INTENSITY_BASED_LocalIntensityPeak(SUVbw)IBSI:VJGA	0.1579
INTENSITY-HISTOGRAM_IntensityHistogramMean(SUVbw)IBSI:X6K6	0.3317
INTENSITY-HISTOGRAM_IntensityHistogramVariance(SUVbw)IBSI:CH89	0.2844
INTENSITY-HISTOGRAM_IntensityHistogramSkewness(SUVbw)IBSI:88K1	0.2666
INTENSITY-HISTOGRAM_IntensityHistogramKurtosis(SUVbw)IBSI:C3I7	0.3625
INTENSITY-HISTOGRAM_IntensityHistogramMedian(SUVbw)IBSI:WIFQ	0.4640
INTENSITY-HISTOGRAM_IntensityHistogramMinimumGreyLevel(SUVbw)IBSI:1PR8	0.4038
INTENSITY-HISTOGRAM_IntensityHistogram10thPercentile(SUVbw)IBSI:GPMT	0.4248
INTENSITY-HISTOGRAM_IntensityHistogram25thPercentile(SUVbw)IBSI:No	0.4411
INTENSITY-HISTOGRAM_IntensityHistogram50thPercentile(SUVbw)IBSI:No	0.4640
INTENSITY-HISTOGRAM_IntensityHistogram75thPercentile(SUVbw)IBSI:No	0.4938
INTENSITY-HISTOGRAM_IntensityHistogram90thPercentile(SUVbw)IBSI:OZ0C	0.4632
INTENSITY-HISTOGRAM_IntensityHistogramStandardDeviation(SUVbw)IBSI:No	0.4981
INTENSITY-HISTOGRAM_IntensityHistogramMaximumGreyLevel(SUVbw)IBSI:3NCY	0.4989
INTENSITY-HISTOGRAM_IntensityHistogramMode(SUVbw)IBSI:AMMC	0.4262
INTENSITY-HISTOGRAM_IntensityHistogramInterquartileRange(SUVbw)IBSI:WR0O	0.4296
INTENSITY-HISTOGRAM_IntensityHistogramRange(SUVbw)IBSI:5Z3W	0.4383
INTENSITY-HISTOGRAM_IntensityHistogramMeanAbsoluteDeviation(SUVbw)IBSI:D2ZX	0.4512
INTENSITY-HISTOGRAM_IntensityHistogramRobustMeanAbsoluteDeviation(SUVbw)IBSI:WRZB	0.2696
INTENSITY-HISTOGRAM_IntensityHistogramMedianAbsoluteDeviation(SUVbw)IBSI:4RNL	0.4225
INTENSITY-HISTOGRAM_IntensityHistogramCoefficientOfVariation(SUVbw)IBSI:CWYJ	0.3506
INTENSITY-HISTOGRAM_IntensityHistogramQuartileCoefficientOfDispersion(SUVbw)IBSI:SLWD	0.3779
INTENSITY-HISTOGRAM_IntensityHistogramEntropyLog10(SUVbw)IBSI:No	0.2184
INTENSITY-HISTOGRAM_IntensityHistogramEntropyLog2(SUVbw)IBSI:TLU2	0.1961
INTENSITY-HISTOGRAM_AreaUnderCurveCsh(SUVbw)IBSI:No	0.1449
INTENSITY-HISTOGRAM_Uniformity(SUVbw)IBSI:BJ5W	0.3360
INTENSITY-HISTOGRAM_RootMeanSquare(SUVbw)IBSI:No	0.0540
INTENSITY-HISTOGRAM_MaximumHistogramGradient(SUVbw)IBSI:12CE	0.3854
INTENSITY-HISTOGRAM_MaximumHistogramGradientGreyLevel(SUVbw)IBSI:8E6O	0.4987
INTENSITY-HISTOGRAM_MinimumHistogramGradient(SUVbw)IBSI:VQB3	0.0529
INTENSITY-HISTOGRAM_MinimumHistogramGradientGreyLevel(SUVbw)IBSI:RHQZ	0.3644

LOCAL_INTENSITY_HISTOGRAM_IntensityPeakDiscretizedVolumeSought(0.5mL)(mL)IBSI:No	0.1986
LOCAL_INTENSITY_HISTOGRAM_GlobalIntensityPeak(0.5mL)(SUVbw)IBSI:No	0.4210
LOCAL_INTENSITY_HISTOGRAM_IntensityPeakDiscretizedVolumeSought(1mL)(mL)IBSI:No	0.1986
LOCAL_INTENSITY_HISTOGRAM_GlobalIntensityPeak(1mL)(SUVbw)IBSI:No	0.2160
LOCAL_INTENSITY_HISTOGRAM_LocalIntensityPeak(SUVbw)IBSI:No	0.4950

Supplementary Table S6: univariate testing of radiomic features according to disease stage.

Variabile	p-val
MORPHOLOGICAL_Volume(IBSI:RNU0)	0.4632
MORPHOLOGICAL_ApproximateVolume(IBSI:YEKZ)	0.1567
MORPHOLOGICAL_voxelsCounting(IBSI:No)	0.2725
MORPHOLOGICAL_SurfaceArea(IBSI:C0JK)	0.9371
MORPHOLOGICAL_SurfaceToVolumeRatio(IBSI:2PR5)	0.0007
MORPHOLOGICAL_Compacity(IBSI:No)	0.6313
MORPHOLOGICAL_Compactness1(IBSI:SKGS)	0.0040
MORPHOLOGICAL_Compactness2(IBSI:BQWJ)	0.0088
MORPHOLOGICAL_SphericalDisproportion(IBSI:KRCK)	0.4239
MORPHOLOGICAL_Sphericity(IBSI:QCFX)	0.0030
MORPHOLOGICAL_CentreOfMassShift(IBSI:KLMA)	0.1956
MORPHOLOGICAL_Maximum3DDiameter(IBSI:L0JK)	0.2758
MORPHOLOGICAL_IntegratedIntensity(IBSI:99N0)	0.6905
INTENSITY-BASED_Variance(SUVbw)IBSI:ECT3	0.9923
INTENSITY-BASED_Skewness(SUVbw)IBSI:KE2A	0.3112
INTENSITY-BASED_Kurtosis(SUVbw)IBSI:IPH6	0.1071
INTENSITY-BASED_Median(SUVbw)IBSI:Y12H	0.8175
INTENSITY-BASED_MinimumGreyLevel(SUVbw)IBSI:1GSF	0.6886
INTENSITY-BASED_StandardDeviation(SUVbw)IBSI:No	0.6333
INTENSITY-BASED_MaximumGreyLevel(SUVbw)IBSI:84IY	0.1752
INTENSITY-BASED_InterquartileRange(SUVbw)IBSI:SALO	0.4282
INTENSITY-BASED_Range(SUVbw)IBSI:2OJQ	0.6298
INTENSITY-BASED_MeanAbsoluteDeviation(SUVbw)IBSI:4FUA	0.7011
INTENSITY-BASED_RobustMeanAbsoluteDeviation(SUVbw)IBSI:1128	0.9486
INTENSITY-BASED_MedianAbsoluteDeviation(SUVbw)IBSI:N72L	0.9301
INTENSITY-BASED_CoefficientOfVariation(SUVbw)IBSI:7TET	0.0194
INTENSITY-BASED_QuartileCoefficientOfDispersion(SUVbw)IBSI:9S40	0.1581
INTENSITY-BASED_AreaUnderCurveCsh(SUVbw)IBSI:No	0.2618
INTENSITY-BASED_Energy(SUVbw)IBSI:N8CA	0.2501
INTENSITY-BASED_RootMeanSquare(SUVbw)IBSI:5ZWQ	0.2588
INTENSITY-BASED_TotalLesionGlycolysis(SUVbw)IBSI:No	0.1167
LOCAL_INTENSITY_BASED_IntensityPeakDiscretizedVolumeSought(0.5mL)(mL)IBSI:No	0.6565
LOCAL_INTENSITY_BASED_GlobalIntensityPeak(0.5mL)(SUVbw)IBSI:No	0.1114
LOCAL_INTENSITY_BASED_IntensityPeakDiscretizedVolumeSought(1mL)(mL)IBSI:No	0.6565
LOCAL_INTENSITY_BASED_GlobalIntensityPeak(1mL)(SUVbw)IBSI:0F91	0.3629
LOCAL_INTENSITY_BASED_LocalIntensityPeak(SUVbw)IBSI:VJGA	0.4785
INTENSITY-HISTOGRAM_IntensityHistogramMean(SUVbw)IBSI:X6K6	0.8883

INTENSITY-HISTOGRAM_IntensityHistogramVariance(SUVbw)IBSI:CH89	0.3230
INTENSITY-HISTOGRAM_IntensityHistogramSkewness(SUVbw)IBSI:88K1	0.4426
INTENSITY-HISTOGRAM_IntensityHistogramKurtosis(SUVbw)IBSI:C3I7	0.5651
INTENSITY-HISTOGRAM_IntensityHistogramMedian(SUVbw)IBSI:WIFQ	0.7143
INTENSITY-HISTOGRAM_IntensityHistogramMinimumGreyLevel(SUVbw)IBSI:1PR8	0.4911
INTENSITY-HISTOGRAM_IntensityHistogram10thPercentile(SUVbw)IBSI:GPMT	0.5007
INTENSITY-HISTOGRAM_IntensityHistogram25thPercentile(SUVbw)IBSI:No	0.5870
INTENSITY-HISTOGRAM_IntensityHistogram50thPercentile(SUVbw)IBSI:No	0.7143
INTENSITY-HISTOGRAM_IntensityHistogram75thPercentile(SUVbw)IBSI:No	0.8127
INTENSITY-HISTOGRAM_IntensityHistogram90thPercentile(SUVbw)IBSI:OZ0C	0.9085
INTENSITY-HISTOGRAM_IntensityHistogramStandardDeviation(SUVbw)IBSI:No	0.0590
INTENSITY-HISTOGRAM_IntensityHistogramMaximumGreyLevel(SUVbw)IBSI:3NCY	0.8093
INTENSITY-HISTOGRAM_IntensityHistogramMode(SUVbw)IBSI:AMMC	0.5468
INTENSITY-HISTOGRAM_IntensityHistogramInterquartileRange(SUVbw)IBSI:WR0O	0.5926
INTENSITY-HISTOGRAM_IntensityHistogramRange(SUVbw)IBSI:5Z3W	0.7594
INTENSITY-HISTOGRAM_IntensityHistogramMeanAbsoluteDeviation(SUVbw)IBSI:D2ZX	0.2735
INTENSITY-HISTOGRAM_IntensityHistogramRobustMeanAbsoluteDeviation(SUVbw)IBSI:WRZB	0.3478
INTENSITY-HISTOGRAM_IntensityHistogramMedianAbsoluteDeviation(SUVbw)IBSI:4RNL	0.1936
INTENSITY-HISTOGRAM_IntensityHistogramCoefficientOfVariation(SUVbw)IBSI:CWYJ	0.0161
INTENSITY-HISTOGRAM_IntensityHistogramQuartileCoefficientOfDispersion(SUVbw)IBSI:SLWD	0.1942
INTENSITY-HISTOGRAM_IntensityHistogramEntropyLog10(SUVbw)IBSI:No	0.3730
INTENSITY-HISTOGRAM_IntensityHistogramEntropyLog2(SUVbw)IBSI:TLU2	0.2679
INTENSITY-HISTOGRAM_AreaUnderCurveCsh(SUVbw)IBSI:No	0.2134
INTENSITY-HISTOGRAM_Uniformity(SUVbw)IBSI:BJ5W	0.6102
INTENSITY-HISTOGRAM_RootMeanSquare(SUVbw)IBSI:No	0.0072
INTENSITY-HISTOGRAM_MaximumHistogramGradient(SUVbw)IBSI:12CE	0.1096
INTENSITY-HISTOGRAM_MaximumHistogramGradientGreyLevel(SUVbw)IBSI:8E6O	0.5305
INTENSITY-HISTOGRAM_MinimumHistogramGradient(SUVbw)IBSI:VQB3	0.6833
INTENSITY-HISTOGRAM_MinimumHistogramGradientGreyLevel(SUVbw)IBSI:RHQZ	0.7619
LOCAL_INTENSITY_HISTOGRAM_IntensityPeakDiscretizedVolumeSought(0.5mL)(mL)IBSI: No	0.6565
LOCAL_INTENSITY_HISTOGRAM_GlobalIntensityPeak(0.5mL)(SUVbw)IBSI:No	0.3212
LOCAL_INTENSITY_HISTOGRAM_IntensityPeakDiscretizedVolumeSought(1mL)(mL)IBSI:No	0.6565
LOCAL_INTENSITY_HISTOGRAM_GlobalIntensityPeak(1mL)(SUVbw)IBSI:No	0.4874
LOCAL_INTENSITY_HISTOGRAM_LocalIntensityPeak(SUVbw)IBSI:No	0.5983
